# Extraspinal findings prevalence and clinical significance in 4250 lumbar spine MRI exams

**DOI:** 10.1038/s41598-021-81069-y

**Published:** 2021-01-13

**Authors:** Ruba A. Khasawneh, Ziyad Mohaidat, Firas A. Khasawneh, Ahmad Farah, Maha Gharaibeh, Mwaffaq El-Heis

**Affiliations:** 1 Department of Diagnostic Radiology and Nuclear Medicine, Faculty of Medicine, King Abdullah University Hospital, Jordan University of Science and Technology, Irbid, 22110 Jordan; 2 Orthopedic Division, Special Surgery Department, Faculty of Medicine, King Abdullah University Hospital, Jordan University of Science and Technology, Irbid, 22110 Jordan; 3 Department of Mechanical Engineering, Michigan State University, East Lansing, MI 48824 USA

**Keywords:** Magnetic resonance imaging, Skeleton

## Abstract

To assess extraspinal findings (ESFs) prevalence in lumbar spine MRI, including clinically significant findings using a systematic approach, and to determine their reporting rate. Lumbar spine MRI scans were retrospectively reviewed over 18 months by two radiologists. Reading discrepancies were resolved by consensus. ESFs were classified according to the involved system, clinical diagnosis, and clinical significance. The reporting rate was estimated by referring to the original report. There were 1509 ESFs in 1322/4250 patients with a substantial agreement between the two radiologists (kappa = 0.8). Almost half (621/1322) were in the 45–60 age group. Females represented 56.6% (748/1322). 74.2% (1120/1509) of the ESFs involved the urinary system among which 79.6% (892/1120) were renal cysts. Clinically significant findings represented 8.7% (131/1509) among which hydronephrosis represented 23% (30/131). First time detected malignant lesions represented 4.6% (6/131). ESFs reporting rate was 47.3%. 58.8% of the clinically significant ESFs were not reported. ESFs prevalence was 31.1%. The Urinary system was the most commonly involved. Most ESFs were benign warranting no further workup. However, clinically significant ESF were not infrequently detected. More than half of the clinically significant findings were not reported. A systematic review of MRI images is highly recommended to improve patient’s outcome.

## Introduction

Lumbar MRI scans are commonly encountered in radiologists’ daily practice. These scans are primarily protocolled for the evaluation of the spinal column and its contents. Various ESFs involving different abdominopelvic organs are frequently detected in the examined field of view.

The introduction of the Picture Archiving and Communication System (PACS) has increased the number of reported ESFs^1–4^. This may unnecessarily increase further investigations and follow up examinations^1–4^. On the other hand, underreporting of ESFs, especially those of clinical significance can adversely affect subsequent patient management. In addition, it can raise ethical and legal concerns for the reading radiologists^3,5,6^.

Several studies have investigated the different aspects of ESFs detected in the lumbar spine MRI including their prevalence, distribution over the different organs, and their reporting rates^3,5–8^. Variable ESFs prevalence rate was reported among different age groups with a range of 9.5–68.8%^3,5,7,9^ The role of localizer images in increasing detection of extraspinal findings as well as the prevalence of incidental extraspinal malignancies in lumbar spine MRI scans had also been investigated^1,10,11^. Two large retrospective cohort studies used a structured approach to identify ESFs and classify them according to their clinical significance based on CT colonography reporting and data system (C-RADS)^3,5^. However, no standard objective method is yet available to determine the clinical significance of the ESFs.

In the literature, there is a well-documented variability in the interpretation of radiological exams^12^. Similarly, the reporting of ESFs in the lumbar spine remains non-uniform and biased by the different radiologists’ practice or personal judgment among other factors. Guidelines specific for the ESFs detected in the lumbar spine MRI are needed to achieve better consistency in the identification and reporting of ESFs, thus contributing to better patient management.

In this study which included a large cohort of Jordanian patients, the different aspects of ESFs are investigated including, their prevalence and distribution according to age, sex, systems involved, and clinical diagnosis. In addition, the clinically significant findings are described. Also, the reporting rates for the different ESFs are estimated.

## Methods

This is a retrospective study performed at the radiology department at King Abdullah University Hospital (KAUH). Patients with lumbar spine MRI scans performed over the period from May 2015 till December 2016 were included in this study. Patients less than 1 year of age, and with a known diagnosis of cancer were excluded from the study. Incomplete exams were also excluded. As for patients who have more than one lumbar MRI scan during the study period; the first chronological study was only included.

An extraspinal finding (ESF), in the present study, is defined as any abnormality detected outside the region for which the scan was primarily performed and is unrelated to the lumbar spine and its contents. Using a standardized data collection form, the ESFs were classified according to the involved organ/system. Then, they were subclassified according to the specific clinical diagnoses in each organ/system as outlined in Table 1. The legend of Table 1 shows the radiological criteria used for some of the clinical diagnoses. Scout images were reviewed, selectively, to verify organomegaly and to assess the distension status of the urinary bladder for accurate measurement of bladder wall thickness and assessment of hydronephrosis.

**Table 1:** The distribution of Extraspinal Findings according to the involved systems and diagnosis.

System	Total		ESF diagnosis	ESF/system			%/Total	Reported		Unreported	
	#	%		#	%		ESFs	#	%	#	%
Urinary	1120	74.2									
system			Renal cyst/s	892	79.6		59.1	524	59	368	41
			Renal scarring/atrophy	62	5.5		4.1	26	42	36	58
			Congenital anomalies	50	4.5		3.3	27	54	23	46
			Hydroureteronephrosis^a^	30	2.7		2.0	20	67	10	33
			Urinary bladder wall thickening^b^	29	2.6		1.9	8	28	21	72
			Single kidney	20	1.8		1.3	11	55	9	45
			Adrenal lesion/s	23	2.1		1.5	4	17	19	83
			Kidney stone/s	7	0.6		0.5	4	57	3	43
			Kidney transplant	4	0.4		0.3	3	75	1	25
			Solid renal mass	3	0.3		0.2	1	33	2	67
Genital	198	13.1									
system			Ovarian cyst/s	87	43.9		5.8	30	34	57	66
			Uterine anomalies	51	25.8		3.4	3	6	48	94
			Uterine fibroids	38	19.2		2.5	4	11	34	89
			Nabothian cyst/s	11	5.6		0.7	0	0	11	100
			Thickened endometrium^c^	10	5.1		0.7	0	0	10	100
			Gravid uterus	1	0.5		0.1	1	100	0	0
Gastrointestinal	110	7.3									
system			Biliary system	55	50		3.6	12	22	43	78
			Liver lesions	43	39.1		2.8	11	26	32	74
			Pancreatic cyst	2	1.8		0.1	1	50	1	50
			Bowel abnormalities	5	4.5		0.3	0	0	5	100
			Organomegaly^d^	5	4.5		0.3	0	0	5	100
Musculoskeletal	19	1.3									
system			Solid masses	6	31.6		0.4	5	83	1	17
			Intramuscular abscess/es	5	26.3		0.3	5	100	0	0
			Intramuscular hematoma	2	10.5		0.1	1	50	1	50
			Muscle atrophy	6	31.6		0.4	3	50	3	50
Vascular	49	3.2									
system			AAA^e^	6	12.2		0.4	6	100	0	0
			Retro-aortic left renal vein	43	87.8		2.8	1	2	42	98
Others	13	0.9									
			Free fluid	9	69.2		0.6	2	22	7	78
			LAP and para-aortic cystic lesions^f^	4	30.8		0.3	1	25	3	75
Total	1509	100					100	714		795	

^#^Number, % percentage, *ESF* extraspinal finding.

^a^Abnormal distension of the calyces as graded per the Society of Fetal Urology (SFU).

^b^Thickness > 3 mm from its outer to inner borders in fully distended status as assessed in scout images.

^c^Thickness > 8 mm postmenopausal.

^d^Splenomegaly and hepatomegaly > 14,16 cm in length respectively.

^e^Abdominal Aortic Aneurysm (transverse diameter > 3 cm).

^f^Lymphadenopathy; short axis > 1 cm.

The scans were reviewed independently by two radiologists with an average of 11 years of experience. ESFs were recorded as present or absent. The final count of the ESFs was determined after resolving any discrepancy in ESF readings between the two radiologists by consensus.

ESFs were also categorized according to their clinical significance. Clinically significant findings are defined as; findings warranting further workup or may affect the patient’s outcome, or those that should be communicated to the treating physician. Non-clinically significant findings included normal variants, benign and mostly benign lesions. Those definitions were derived based on the accepted practice guidelines, the ACR’s Incidental Findings Committee II recommendations, literature, and authors’ consensus^13–15^.

The reporting rate of ESFs, including both clinically and non-clinically significant findings, was estimated after the retrospective review of the archived MRI reports which were signed off by 9 different KAUH radiologists with 10–30 years of experience following double readings of residents’ reports. The two radiologists who performed the present study were blinded to the archived report at the initial review of the MRI scans.

The lumbar spine MRI scans were performed on a 1.5 T MRI machine (Ingenia, Philips) using the spine coil integrated in the patient’s table. The MRI study protocol included: 1-Sagittal T1 weighted fast spin-echo (TR/TE 400–600/12 ms, matrix, 180 X301; FOV, 27 cm; 2-Sagittal T2 weighted fast spin-echo (TR/TE 2500/100 ms), matrix, 180 X 335, FOV, 27 cm, and 3-axial T2 weighted fast spin-echo (TR/TE 4880/120 ms), matrix, 180 X335, FOV 20 cm. 4 mm slice thickness with a 0.4-mm interslice gap was used for all sequences. The axial section images were taken between L1 and S1 vertebra. Additional post-contrast images were obtained in some patients with a prior history of spine surgery.

R statistical software package (version 4.0.3)^16^ was used for statistical analysis and management of research data. The inter-observer reliability between the two radiologists was measured using Cohen’s Kappa coefficient. Cohen’s Kappa results are interpreted as follows^17^: values ≤ 0 as indicating no agreement and 0.01–0.20 as none to slight, 0.21–0.40 as fair, 0.41–0.60 as moderate, 0.61–0.80 as substantial, and 0.81–1.00 as almost perfect agreement.

The institutional review board and research committee at the Jordan University of Science and Technology approved this retrospective study and waived the need for informed consent. All methods were performed in accordance with their relevant guidelines and regulations.

## Results

Lumbar MRI scans of 4250 patients were included in the study. The average age of these patients was 55.24 years (age range 1–91 years). Females represented 54.4% (2311/4250) of these patients while males represented 45.6% (1939/4250).

ESFs were detected in 31.1% (1322/4250) of the patients. There was a substantial agreement in the identification of the ESFs between the two radiologists (Kappa = 0.8, 95% Confidence interval = − 0.42 to 1.18), P-value < 0.005). These patients were found to have one or more ESF with a total of 1509 findings. 87.5% of the patients had one ESF (1157/1322), while 11% (145/1322) had two findings. The remaining 1.5% (20/1322) had 3 or more findings. Almost half (705/1509) of the ESFs were seen in patients between 45 and 60 years of age who represented 47% (621/1322) of the patients. ESFs were more commonly seen in females 56.6% (748/1322).

ESFs involved different systems including; the urinary, the genital, the gastrointestinal, the musculoskeletal, the vascular system, and others (Table 1). The urinary system represented the most frequently involved system (74.2%) (Table 1). ESFs in each system were given a specific diagnosis (Table 1). Among all ESFs, the renal cyst was the most common representing 59% while all other findings represented the remaining 41%.

**Figure 1:**
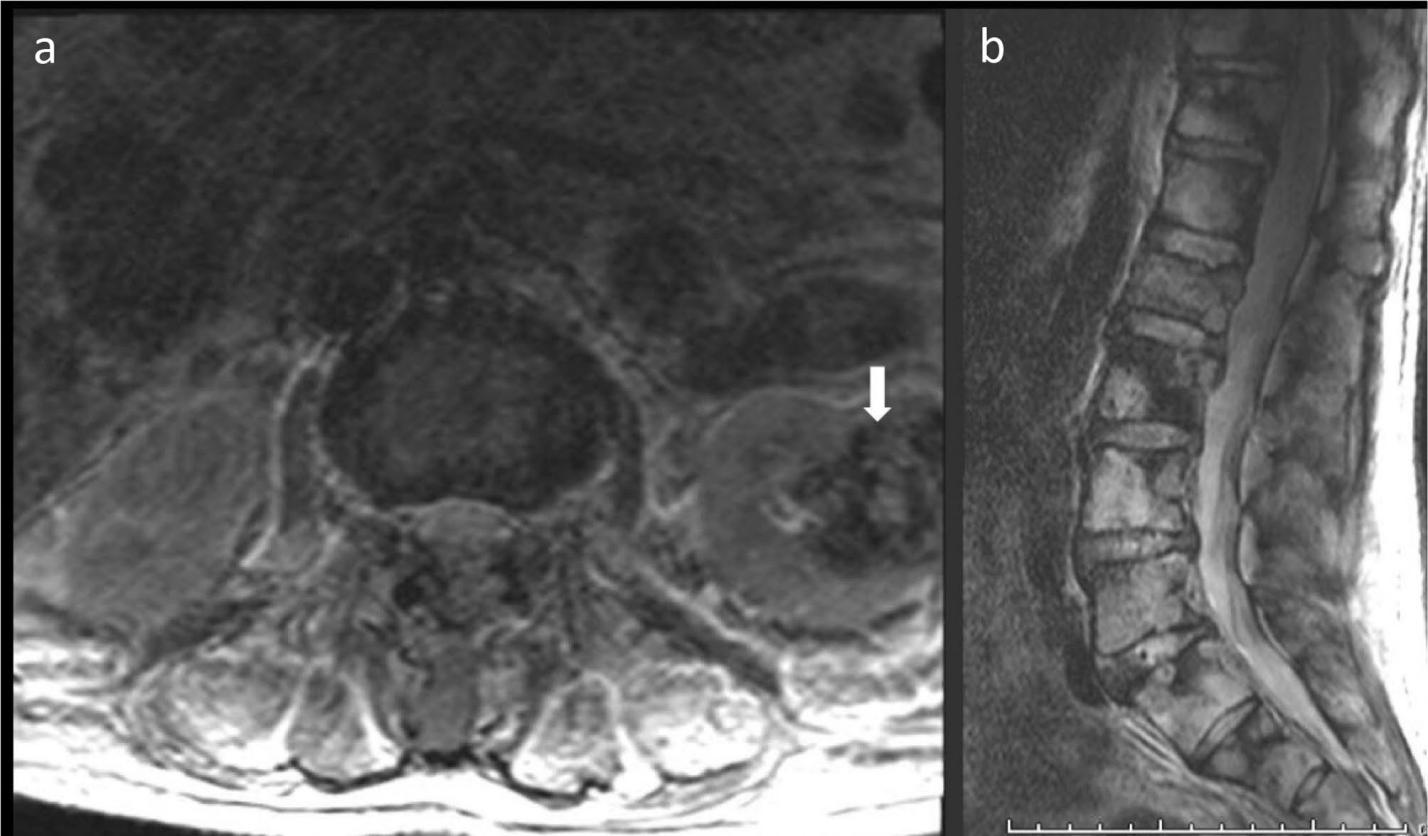
A 75-year-old male patient with left renal cell carcinoma and spine metastasis. (**a**) Axial T2 weighted images showing a heterogenous solid left renal mass (white solid arrow). (**b**) Sagittal T2 weighted images showing diffuse spinal metastasis.

In the urinary system, renal cyst diagnosis included both cortical and parapelvic renal cysts. In addition, unilateral, bilateral, simple, complex, or cysts as part of autosomal dominant polycystic kidney disease (ADPCKD) were considered as one finding in each patient. The majority (861/892) of the cysts were simple renal cysts (Bosniak I). The remaining were Bosniak II, including five cases of ADPCKD. Renal scarring/atrophy and congenital anomalies were almost equally common (Table 1). Congenital anomalies included pelvic/mal-rotated kidney (30/1509), and horseshoe kidney (18/1509). In addition, one pelvic crossed fused renal ectopia was detected in a 32-year-old male and another left multi-cystic dysplastic kidney was seen in a 2-year-old male. Solid renal masses included: angiomyolipoma and two other suspicious heterogenous masses (Fig. 1a,b).

The genital system was the second most common system to have ESFs. Ovarian cysts represented almost half (44%) of these findings (Table 1). They included unilateral or bilateral ovarian cyst/s as well as simple or complex ovarian cysts. Features of cyst complexity included hemorrhagic cyst and cyst with internal septations or soft tissue component. The size of the cyst could not be included in these criteria as most of the cysts were partially visualized on the examined field of view. Complex ovarian cysts represented 17.2% (15/87) of the cysts, while the rest represented simple, mostly physiological or functional ovarian cysts. Retroverted uterus was the most commonly (49/51) encountered as a congenital anomaly of the genital system. Uterine fibroids were the third most common findings within the genital system. They included; single or multiple uterine fibroids whether sub-serosal or intramural in location, and whether with or without degeneration or calcifications.

In the GI system, ESFs of the biliary system were the most common including gallstones (45/55), bile duct dilatations (equal or more than 0.8 cm) with or without stones (8/55), and post cholecystectomy collection (1/55). Most (41/43) of the liver lesions encountered were benign homogenous T2 hyperintense lesions (cysts/hemangiomas) and were mostly encountered in segment VI, which is the portion that is usually covered in the exam. One of the detected liver cysts was mostly consistent with healed hydatid cyst due to associated wall calcifications.

**Figure 2:**
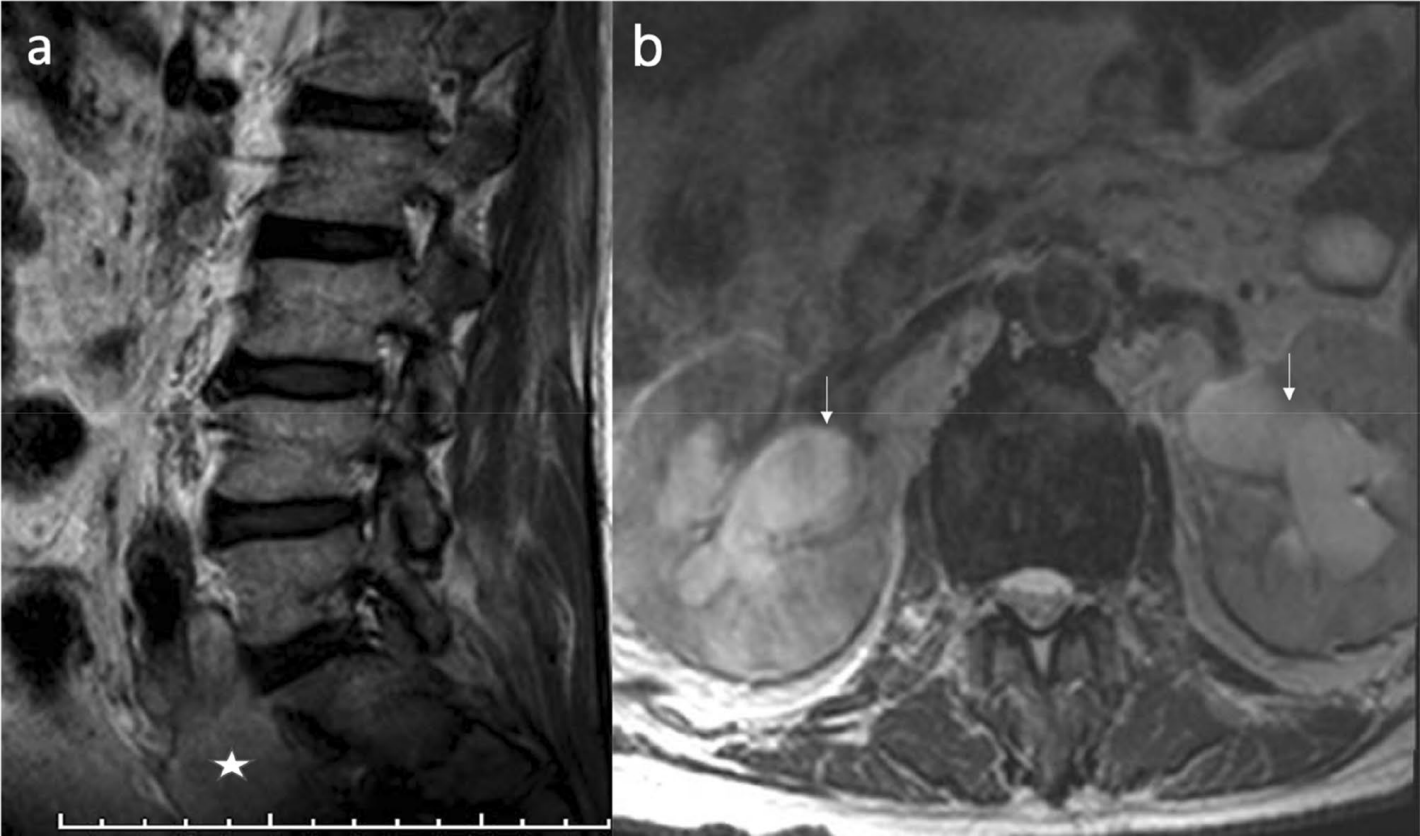
A 65-year-old male with metastatic bladder cancer. (**a**) Sagittal T2 weighted image showing pathological lymph nodes along the left iliac vessels (asterisk). (**b**) Axial T2 weighted image showing bilateral hydronephrotic changes (white arrows).

The clinically significant ESFs, according to the definition in this study, represented about 8.7% (131/1509) of the findings among which hydronephrosis and urinary bladder wall thickening were almost equally common as they represented about 23% (30/131) and 22% (29/131) respectively (Table 2). Hydronephrosis was assessed by the SFU (Society of Fetal Urology) grading system^18^. All the cases included were grade II or more. Cases with minimal calyceal distension probably related to physiological hydronephrosis or overdistension of the urinary bladder as evaluated by scout images were not considered as an ESF. Extrarenal pelvises were not reported as well in this study. In only 6 of the 30 patients with hydronephrosis, the cause of the hydronephrosis was identified on the reviewed MRI images. Among those patients, 2 patients had obstructive uropathy secondary to obstructing pelvi-ureteric stone and distal ureteric stone. In another 2 patients, it was most probably related to a neurogenic bladder as it was associated with thickened trabeculated urinary bladder wall and hydro-uretero-nephrotic changes. Another 65-year-old male with bilateral hydroureteronephrosis, metastatic lymph nodes along the left iliac group were observed. Subsequent workup revealed the diagnosis of bladder cancer (Fig. 2a,b). The lymphadenopathy was not reported in the MRI archived report and the bladder cancer was not covered in the examined field of view. Also, there was one pregnant patient that was diagnosed with right-sided hydronephrotic changes. Medical records review of this patient revealed MRI was performed to evaluate her acute onset lower back pain. Hydronephrosis in this patient can well explain her clinical presentation. Among the 24 patients with no identifiable cause on their MRI scan, subsequent CT stone protocol revealed 2 patients with distal obstructing ureteral stones.

**Table 2:** The distribution of the clinically significant extraspinal findings and their reporting rates.

Systems involved	Findings/system		ESF (Clinically significant)			Reported findings	
	#	%		#	%	#	%
Urinary	70	53					
			Hydronephrosis	30	22.9	20	66.7
			Thick urinary bladder wall	29	22.1	8	27.6
			Kidney/ureteric stone with/without HUN	8	6.1	5	62.5
			Solid renal masses	3	2.3	1	33.3
Gynecological	25	19					
			Complex ovarian cyst	15	11.5	5	33.3
			Thickened endometrial stripe	10	7.6	0	0
Gastrointestinal	13	10					
			Common bile duct stones	4	3.1	0	0
			Suspicious liver pathologies	2	1.5	0	0
			Bowel pathologies				
			Mucocele	1	0.8	0	0
			Dilated bowel with transient intussusception	1	0.8	0	0
			Organomegaly	5	3.8	0	0
Musculoskeletal	9	7					
			Intramuscular abscess	5	3.8	5	100
			Intramuscular hematoma	2	1.5	1	50
			Intramuscular solid mass	2	1.5	2	100
Vascular	6	5					
			Abdominal Aortic Aneurysm	6	4.6	6	100
Others	8	6					
			Ascites	4	3.1	0	0
			Cystic lesions	2	1.5	0	0
			Lymphadenopathy	2	1.5	1	50
Total	131	100		131	100	54	41.2

*ESF* extraspinal findings, #: number, %: percentage, *HUN* hydroureteronephrosis.

**Figure 3:**
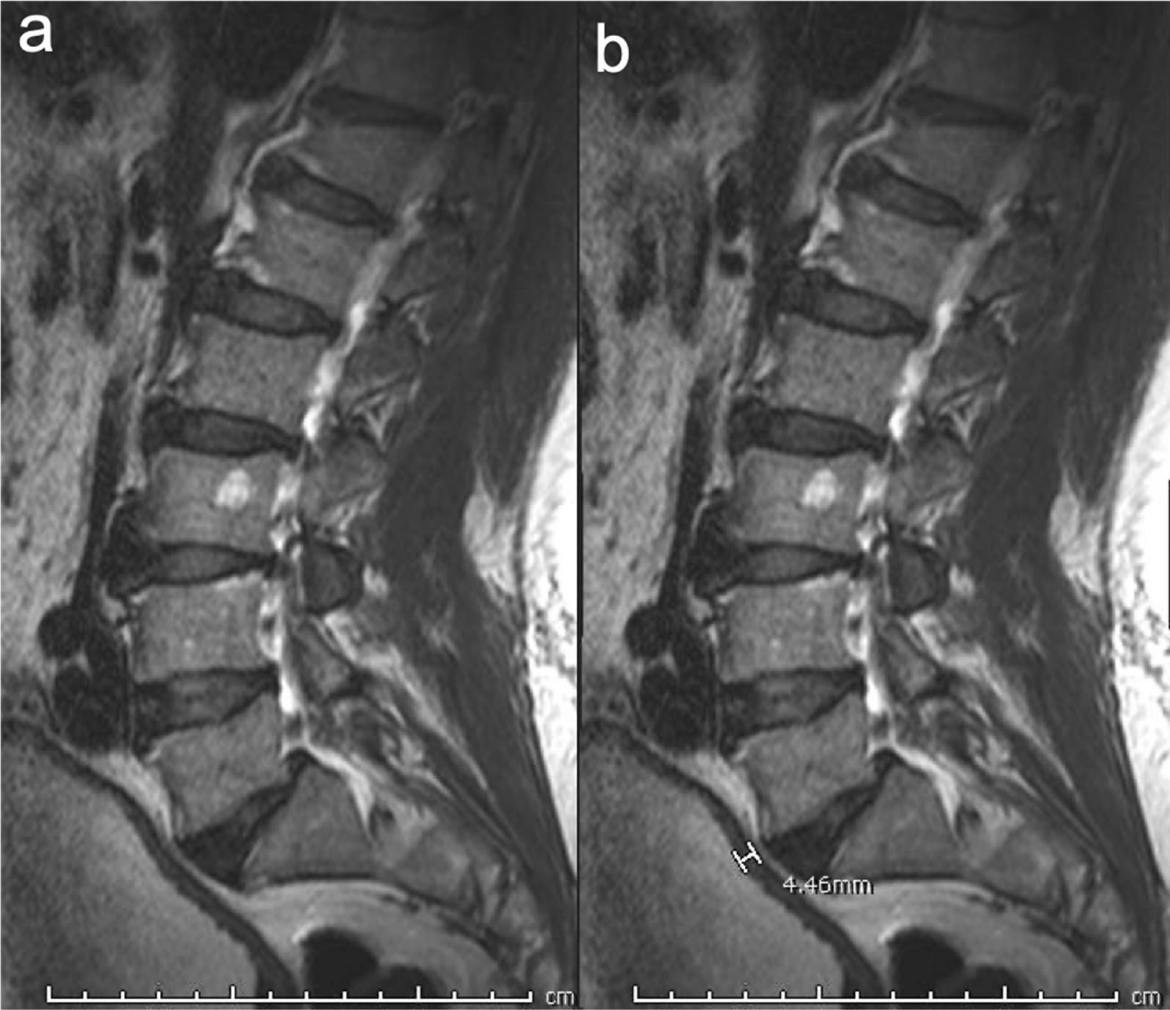
A 79-year-old male patient with thickened urinary bladder wall. (**a**) Sagittal T2 weighted image demonstrating thickening in the posterior wall of the urinary bladder which is well distended and partially visualized. (**b**) Sagittal T2 weighted image at this level with measurement annotation from the outer to the inner borders of the urinary bladder wall showing that it measured more than 4 mm.

Urinary bladder wall thickness was measured from the outer to the inner borders of the posterior wall on the sagittal T2 WI’s (Fig. 3a,b). Since wall thickness can vary based on the distension status of the bladder, scout images were used to assess the distension status. Only fully distended bladders with a wall thickness of more than 3 mm were included. Among those who had subsequent workup, a cause was identified in 13 of the 29 patients. The diagnoses in these patients were BPH, recurrent urinary tract infection, and neurogenic bladder in 5, 3, and 5 patients respectively.

Complex ovarian cysts were the third most common clinically significant ESF. Among the 15 cases detected in this study, 3 had further investigations at our institution. Histopathology revealed endometrioma, cystadenoma, and para-ovarian benign cyst.

In the MSK system, intramuscular abscess, either single or multiple, was the most common clinically significant finding (Table 2). Among the clinically significant solid masses involving the MSK were two intramuscular metastatic lesions from lung cancer in 2 different patients.

The reporting rate of the ESFs was determined after reviewing the archived reports. The reporting rate for the total ESFs was 47.3% (714/1509). Among the clinically significant findings, 58.8% (77/131) were not reported. The clinically significant ESFs involving the vascular and the MSK system were highly reported (Table 2) while the other systems showed a lower reporting rate (Table 2).

All clinically significant findings within the GI system were unreported (Table 2). Among those, there was a 49-year old female who showed changes of diffuse liver cirrhosis. A proven diagnosis of sarcoidosis was confirmed later. A 59-year old female with splenomegaly was diagnosed with liver cirrhosis 3 months later Another 53-year-old male showed multiple liver lesions in association with retroperitoneal LAP; consistent with liver metastasis. He was not known to have malignancy and no previous images were available at the time of the exam. Histopathological examination of one of his liver lesions showed metastatic poorly differentiated lung adenocarcinoma. The primary lung cancer was evident on CT CAP done after the MRI scan.

**Figure 4:**
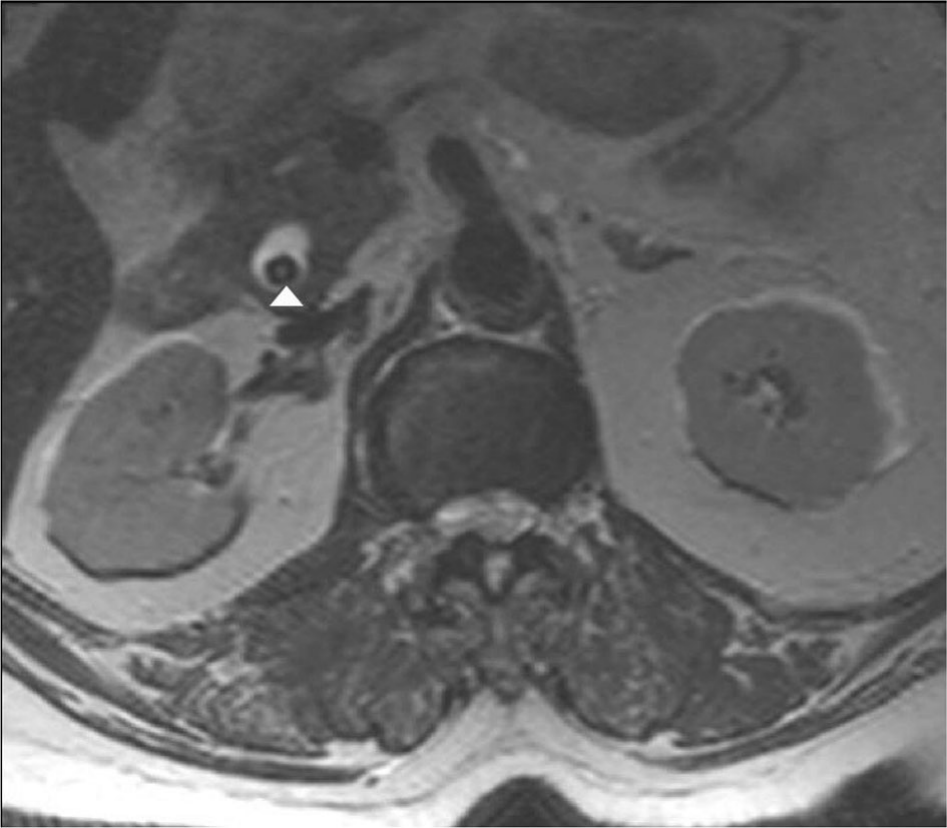
A 68-year-old female patient showing dilated CBD with distal CBD stone (arrow head) in this axial T2 weighted image.

As for bile duct dilatations; 2 patients had common bile duct (CBD) stones in association with dilatation (Fig. 4). Another patient had intra- and extrahepatic biliary dilatation due to benign stricture at the ampulla of Vater as revealed by further investigations. A postoperative seroma/biloma was seen in the gallbladder bed in a 76-year female who had a recent cholecystectomy at an outside facility.

**Figure 5:**
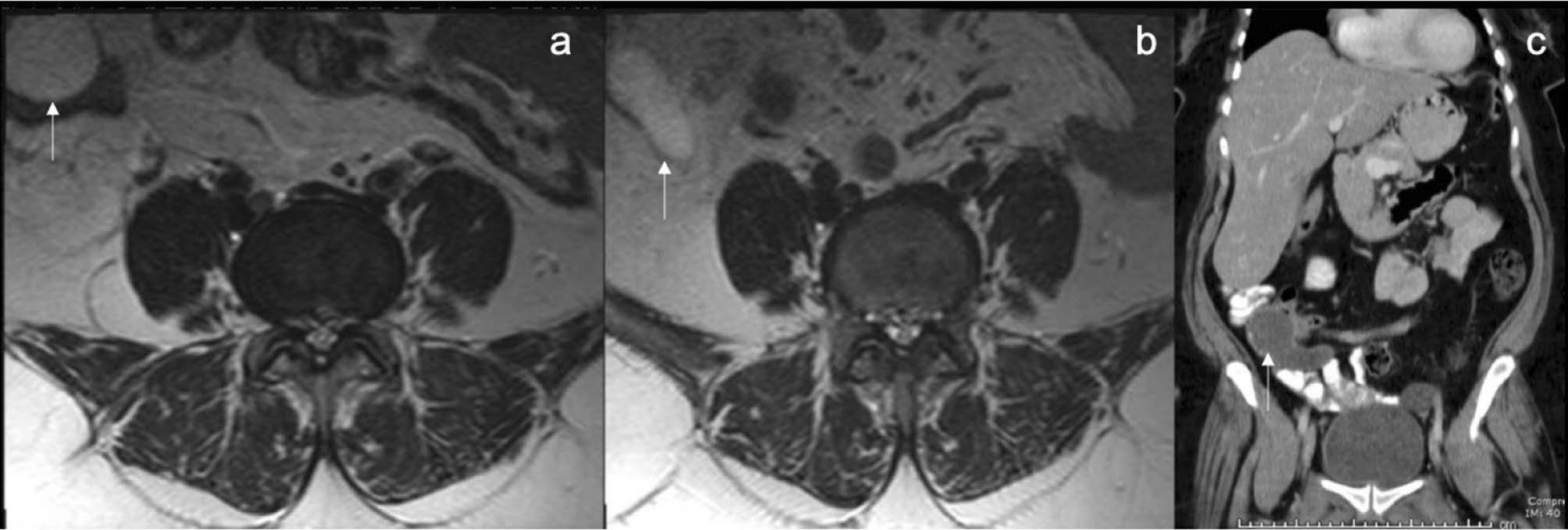
A 49-year-old female with appendiceal mucocele. (**a**) Axial T2 weighted image showing a cystic lesion in relation to the cecum (white arrow). (**b**) Axial T2 weighted image at a lower level showing the tubular shape of the cystic lesion (white arrow). (c) Coronal CT scan reconstruction with oral and IV contrast performed 2 years later demonstrated the same cystic lesion in relation to the cecum with faint wall calcifications (white arrow).

The unreported appendiceal mucocele was partially visualized. In a CT scan of the abdomen and pelvis, performed 2 years later for suspected obstructing kidney stone, the mucocele was fully visualized and was subsequently resected (Fig. 5a–c).

Among the unreported clinically significant ESFs detected in this study, 6 patients showed findings in the examined lumbar MRI scans that were suspicious for malignancy (Table 3).

**Table 3:** Extraspinal findings proved to be related to malignancy upon further investigations.

Age	Sex	Extraspinal finding	Final diagnosis
61	Female	Heterogenous right kidney mass & bone metastasis	Breast cancer with bone metastasis. The kidney mass has never been investigated
75	Male	Heterogenous left kidney mass & bone metastasis	Clear cell renal cell carcinoma
65	Female	Hepatosplenomegaly	Myelofibrosis
53	Male	Heterogenous liver lesions & retroperitoneal lymphadenopathy	Lung cancer
65	Male	Pathological left iliac lymph nodes and bilateral hydroureteronephrosis	High grade urothelial papillary carcinoma of the urinary bladder
37	Male	Splenomegaly	Polycythemia Rubra Vera

Upon review of their medical records, a confirmed diagnosis of malignancy was documented. (Table 3). These findings represented 7.8% (6/77) of the unreported but clinically significant ESFs.

## Discussion

MRI scans of the lumbar spine are commonly performed in daily practice for the evaluation of different pathological conditions. The MRI sectional images obtained in different planes can include variable sections of the extraspinal organ systems. Several studies have discussed the different findings that might be seen in these structures.

This series, with a larger sample size compared to the previous series, showed that the prevalence of ESFs is 31.1%. This prevalence was close to several other studies^5,6,8^. However, it was lower than that reported by Quattrocchi et al.^3^. This variation is mostly due to their MRI protocol covering a larger field of view. This is further supported by their detection of more ovarian and uterine lesions in addition to the free pelvic fluid when compared to other series (Table 4).

**Table 4:** Summary of ESFs reported in previous series.

Extraspinal finding		Tuncel et al.^7^		Zidan et al.^6^		Seeman et al.^5^		Quattrocchi et al.^3^		Current study	
# Patients		1278		379		3024		3000		4250	
# ESFs		253		90		859		2060		1509	
Prevalence%		19.8		23.7		22		68.6		31.1	
Reporting rate (%)		28				59.6		11		47.3	
		**#**	%	#	%	#	%	#	%	#	%
**Urinary system**											
Renal cyst/s		83*	32.8	39	43.3	532	61.9	732	35.5	892	59.1
Renal scarring/atrophy		3	1.2	2	2.2	20*	2.3			62	4.1
Congenital anomalies		4	1.6	1	1.1	10*	1.2			50	3.3
Hydroureteronephrosis		2	0.8	11	12.2	11*	1.3	4	0.2	30	2.0
UB wall thickening		4	1.6	1	1.1	1	0.1	15**	0.7	29	1.9
Single kidney						12	1.4			20	1.3
Adrenal lesion/s		11	4.3			9	1	1	0.05	23	1.5
Kidney stone/s						1	0.1	2	0.1	7	0.5
Kidney transplant						9	1			4	0.3
Solid renal mass		2	0.8			10*	1.2	4	0.2	3	0.2
Prostatic enlargement				4	4.4	6	0.7				
**Genital system**											
Ovarian cyst/s		28	11.1	4	4.4	30	3.5	221	10.7	87	5.8
Uterine anomalies		1	0.4	10	11.1	2	0.2	12	0.6	51	3.4
Uterine fibroids		40	15.8	10	11.1	21	2.4	338	16.4	38	2.5
Nabothian cyst/s				5	5.6	1	0.1			11	0.7
Thickened endometrium		6	2.4	3	3.3	1	0.1	15	0.7	10	0.7
Gravid uterus										1	0.1
**Gastrointestinal system**											
Biliary system		2	0.8			51*	5.9	11*	0.5	55	3.7
Liver lesions		3*	1.2			31*	3.6	33*	1.6	43	2.9
Pancreatic lesions						2	0.2			2	0.1
Bowel abnormalities										2	0.1
Organomegaly						1	0.1			5	0.3
Diverticulosis		1	0.4			3	0.3	351	17	3	0.2
Bowel wall thickening								7	0.3		
**Musculoskeletal system**											
Solid masses						1	0.1			6	0.4
Intramuscular abscess/es										5	0.3
Intramuscular hematoma										2	0.1
Muscle atrophy										6	0.4
**Vascular system**											
Abdominal aortic aneurysm		2	0.8			25	2.9	11	0.5	6	0.4
Vascular anomalies		52	20.6							43	2.9
**Others**											
Free fluid		1	0.4			4*	0.5	204	9.9	9	0.6
LAP & cystic lesions		7	2.8			6*	0.7	38	1.8	4	0.3
		253		90	99.8	803	93.5	2001	97.1		

^#^Number, *ESFs* extraspinal findings. %: percentage, *UB* urinary bladder, *LAP* lymphadenopathy.

*Findings were summed up based on our clinical diagnosis.

**Included prostatic lesion per author's classification for comparison.

ESFs in this study were more common in the age group of 45–60 years which is similar to other studies^6,8^. This age group can represent a common age group for different degenerative changes. The slight female predominance in this series was similar to that reported before^3,5,7^.

Regarding the systems involved by the ESFs, the urinary system was the most common system involved with renal cysts being the most common finding. Other series reported similar findings^3,5–7^. Congenital anomalies of the combined genital and urinary systems constituted about 6.6% of the ESFs, which is higher than that reported by other series^3,5,7^. In the GI system; the prevalence of liver lesions was consistent with other large Cohort studies^3,5^.

**Figure 6:**
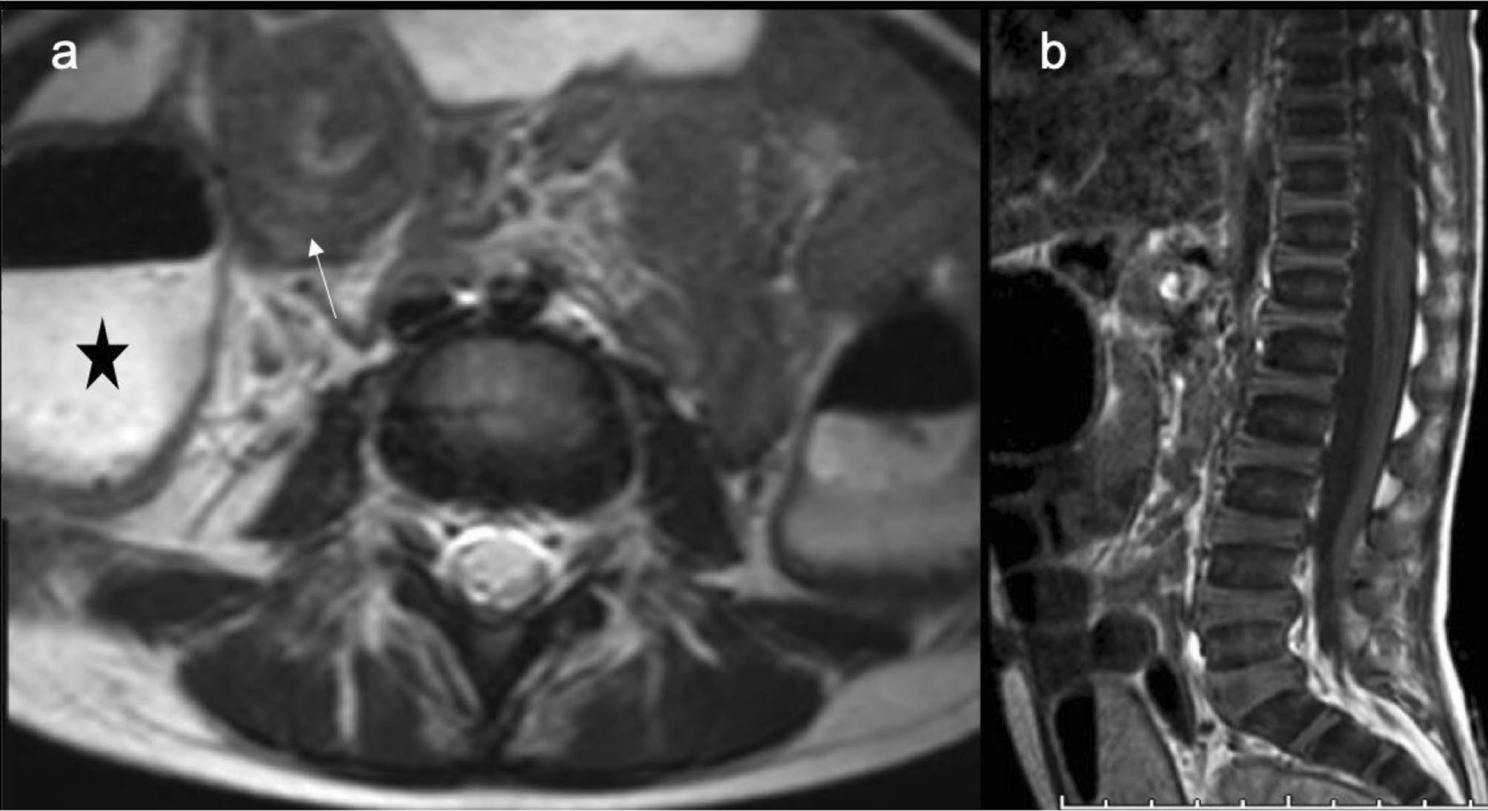
A 3-year-old male patient with Acute lymphocytic leukemia. (**a)** Axial T2 Weighted images showing dilated bowel loops (asterisk) with transient small bowel intussusception (white arrow). (**b)** Sagittal T1 Weighted images, note the diffuse bone marrow replacement. This patient was diagnosed latter with acute lymphocytic leukemia.

The larger sample size included in this series reflected on the diversity of detected ESFs. This is evident by the ESFs involving the MSK as well as some ESFs in the GI systems. The MSK ESFs included two intramuscular metastatic lesions due to lung cancer in 2 different patients. No previous similar series detected such a rare ESF. In the GI system, one case of dilated bowel loops with multiple small bowel intussusceptions (Fig. 6a,b), and another case of appendiceal mucocele (Fig. 5a–c) were interestingly detected solely in this series. On the other hand, only 3 cases of colonic diverticulosis were diagnosed in this series. Although other authors reported a similar very low rate of such a finding^5,7^, Quattrocchi et al.^3^ reported a rate of 20.4% for the same finding. Younger age group, different imaging techniques, and different investigated populations might be contributing factors for this variation.

Hydronephrosis represented about one-fourth of the clinically significant findings, while it represented about 2% of the total ESFs. Other studies reported a variable prevalence of hydronephrosis ranging from 0.2 to 12.2%^3,5–7^ (Table 4). Similar to other series^3,5^; it was considered as a clinically significant ESF since clinical correlation and further workup may be required to identify its possible causes that are usually undefined on lumbar spine MRI. As signified in this series, further workup of patients with hydronephrosis revealed different serious underlying pathologies including one patient with metastatic bladder cancer.

Urinary bladder wall thickness (UBWT) represented about 1.9% of the total ESFs, This rate is close to that reported by Tuncel et al.^7^, however, it is higher than that reported by other series^3,5,6^ (Table 4). This variation can be related to the lack of a standardized definition and methods of measurement of the urinary bladder wall thickness on lumbar spine MRI. The distension status of the urinary bladder during the MRI exam and the different MRI techniques can be considered as other contributing factors. Similar to other studies; UBWT was considered as a clinically significant finding; representing about one-fourth of the clinically significant ESFs in this series. Urinary bladder wall thickness may harbor serious pathologies. This is revealed by the detection of transitional cell bladder cancer in 4 out of the 15 patients who were further investigated for UBWT in Quattrocchi et al. series^3^. Although based on CT scan, another large series^19^ that have investigated the clinical significance of incidentally detected bladder wall thickening; 6.6% (11/167) of these patients were diagnosed with bladder cancer upon subsequent cystoscopy.

Urolithiasis represented 0.5% of the total ESFs. It was a cause of obstructive hydronephrosis in 2 patients in this series. This low detected rate of urolithiasis can be explained by the incomplete coverage of the urinary tract in the examined field of view in addition to the known MRI lower sensitivity in detecting urolithiasis compared to CT scan^3^.

AAA, among the clinically significant ESFs, was uncommonly detected in this study which is similar to other studies^3,8^ (Table 4). On the other hand, AAA as an ESF in lumbar CT for low back pain evaluation was detected at a higher rate^20,21^. This difference can be due to the inherent limitations of MRI for the evaluation of AAA when compared to CT as well as the use of saturation bands on lumbar spine MRI.

Malignant suspicious lesions were infrequently detected in this series, which is similar to other series^1^, representing about 0.5%. As reported before; solid renal masses and lymphadenopathy were among the most common in this category^1,5,7,20^. In this series; these findings represented 7.8% of the unreported clinically significant findings. Given their significant impact on a patient’s prognosis, documentation of such clinically significant ESFs is crucial. This is further supported by the higher survival rates of incidentally detected renal cell carcinoma as observed by Konnak et al.^22^. Xiong et al.^23^ has reported that the prevalence of incidentally detected extracolonic cancer in CT colonography examinations was close to the incidence of cancer detected by some of the specific cancer screening methods. Therefore, a thorough systematic review of lumbar MRI scan with increased attention to the extraspinal structures is highly recommended.

The reporting rate of the ESFs in this study was close to that reported by Semaan et al.^5^ (Table 4); while it was higher than that detected in other series^3,7,8^ (Table 4). However, many clinically significant findings were still underreported (Table 2). It can be noted that ESFs in organ/systems in close anatomical relation to the lumbar spine, including the vascular and the MSK systems, were highly reported. This can reflect that the radiologist’s attention is focused primarily on spinal pathologies and adjacent structures. When compared to other similar large series; the unreported rate of clinically significant findings in this series (58.8%) was less than that reported by Quattrocchi et al. (85%)^3^ while it was higher than that reported by Semaan et al. (38.6%)^5^. This variability can lead to confusion among treating physicians. In fact, it is hard to know the real impact of a clinically significant ESF without follow up of patients’ subsequent investigations and outcomes. This study has revealed the clinical significance of a variety of unreported ESFs in terms of patients’ outcome. Such feedback is important in enhancing radiologists learning and developing evidence-based image guidelines as well as departmental quality assurance programs to minimize potential harms for the patients.

The variable reporting rate of ESFs in lumbar spine MRI among different series including this series can be attributed to several factors including radiologist experience, lack of complete clinical information at the time of reporting, work volume, and MRI protocol variations among different institutions. In addition, the radiologists might also be selective in reporting ESFs based on their clinical judgment. Underreporting of ESFs can be also attributed to the Satisfaction of Search (SOS)^24^; which is a common error in diagnostic radiology. Once a radiologist identifies an abnormality in the radiological exam; other lesions remain undetected. While reporting benign non-clinically significant findings might increase the impact on the health system and unnecessarily increase patient’s anxiety^1,2^; Underreporting clinically significant findings will raise practical and ethical issues for the reading radiologist^5^. Also, it is hard to provide a conclusive benign assumption for the diagnostically indeterminate ESFs without further investigations in an attempt to reach a certain diagnosis. Thus, we believe that all ESFs should be included in the radiology report with further elaboration on their clinical significance to avoid unnecessary interventions.

The authors believe that the C-RADS adopted by some authors does not contribute to increasing the detection rate of the ESFs. Furthermore; the clinical importance for the same ESFs, using the C-RADS, has been assigned differently among different researchers^20^. Thus, further research is needed to establish a reporting system or structured radiology report specific for the lumbar spine MRI taking into consideration a clear definition for the disease categories commonly seen in lumbar spine MR with practical guide for follow up and management based on current recommendations.

Some limitations of this study include its retrospective design and the unavailability of patient’s follow up. This might have contributed to underestimation of the real impact for some of the assigned clinically significant ESFs in the studied group. The original reports were read by different independent radiologists with variable levels of experience which might have contributed to variation in reporting of ESFs. On the other hand, this study reflects the real practice in most institutions where lumbar MRI scans are reported by radiologists with different subspecialties and cumulative years of experience.

## Conclusion

ESFs, in this study, were more common in patients of middle age and diagnosed slightly more in females than males. Using the systematic approach; the prevalence of ESFs was about 31.1% with a substantial agreement between the reading radiologists in detecting ESFs. ESFs involved multiple systems with the urinary system being the most common. Renal cyst/s were the most common ESF. Most ESFs were benign in nature warranting no further investigations or follow up. However, clinically significant ESF represented 8.7% of the findings. Hydronephrosis was the most common clinically significant finding. ESF reporting rate was about 47.3%. ESFs involving the musculoskeletal and the vascular systems were highly reported. More than half of the clinically significant findings were not reported among which 6 patients had a proven malignancy upon further investigations. Therefore, a systematic review of MRI images in all performed planes is highly recommended since it can contribute to early detection and reporting of clinically significant asymptomatic ESFs on lumbar spine MRI. This approach can ultimately improve patient management and outcome.

## Data Availability

The datasets generated during and/or analyzed during the current study are available from the corresponding author on reasonable request.
